# Psychometric properties of the transgender identity stigma scale in Argentina

**DOI:** 10.3389/fpsyg.2025.1531749

**Published:** 2025-05-02

**Authors:** Jeffrey Wilmer Ramos-Santiago, Romina Caballero, Virginia Zalazar, Pablo D. Radusky, Nadir Cardozo, Boris Brunori, Alixida Ramos-Pibernus, Ana Paula Cupertino, Raúl Mejía, Francisco Cartujano-Barrera, Inés Aristeguí

**Affiliations:** ^1^Department of Public Health Sciences, University of Rochester, Rochester, NY, United States; ^2^Department of Surgery, University of Rochester, Rochester, NY, United States; ^3^Research Department, Fundación Huésped, Buenos Aires, Argentina; ^4^Faculty of Psychology, Universidad de Buenos Aires, Buenos Aires, Argentina; ^5^School of Behavioral and Brain Sciences, Ponce Health Sciences University, Ponce, Puerto Rico

**Keywords:** transgender identity, stigma, transgender and gender-diverse, psychometric, scale development

## Abstract

**Introduction:**

Transgender identity stigma (TIS) threatens the well-being of transgender and gender-diverse (TGD) individuals. To the best of our knowledge, there are no validated TIS measures developed for TGD individuals living in Argentina. The purpose of this study was to evaluate the psychometric properties of a TIS scale among a sample of TGD individuals living in Argentina.

**Methods:**

This study consisted of a secondary data analysis of the TransCITAR cohort study. Participants were 484 TGD individuals living in Buenos Aires, Argentina. Items from the HIV Stigma Index and the Everyday Discrimination Scale were combined and adapted to design the TIS scale. Item reduction analyses were carried out. Keyser-Meyer-Olkin (KMO) test of sampling adequacy and Bartlett’s Test of Sphericity were examined to verify the factorability of the scale. Exploratory factor analyses (EFA) were conducted using a principal component method with promax rotation to identify the underlying factors of the scale.

**Results:**

The KMO value was 0.83 and the Bartlett’s Test of Sphericity showed correlations between the variables (χ2 = 5,901.26, d.f. = 66, *p* < 0.001). Upon demonstrating factorability, an EFA was calculated with the 12 items selected for the final version of the TIS scale. Two factors were extracted, explaining 72% of the total variance.

**Conclusion:**

The TIS scale showed great internal consistency, reliability, and construct and criterion validity among TGD individuals living in Argentina, with applicability in both research and clinical settings.

## 1 Introduction

Stigma occurs when individuals and/or institutions label, stereotype, and reject groups of people, which keeps them from accessing social, economic, and political power (King et al., 2020). In this study, transgender identity stigma (TIS) refers to stigma faced by transgender and gender-diverse (TGD) individuals related to their gender identity, which can be enacted and internalized (Moallef et al., 2022). Enacted TIS involves enacted mistreatment and rejection from others (Veale et al., 2017), which can then be internalized and result in shame and guilt, anticipation of rejection, and self-exclusion (Austin and Goodman, 2017). In turn, TIS leads to many TGD individuals being excluded from their homes and educational institutions, resulting in multiple social vulnerabilities (e.g., limited employment opportunities, financial distress, unstable housing, engagement in sex work) (King et al., 2020; Moallef et al., 2022; Veale et al., 2017; Austin and Goodman, 2017; Winter et al., 2016; Radusky et al., 2021; Zalazar et al., 2018; Radusky et al., 2022) TIS threatens the well-being of TGD individuals on a global scale, and Argentina is no exception (Ministerio Público de la Defensa de la Ciudad Autónoma de Buenos Aires & Bachillerato Popular Trans Mocha Celis: la revolución de las mariposas, 2017; Censo Nacional de Población Hogares Viviendas, 2022; Fundación Huésped: gender identity law and transgender people access to health care in Argentina, 2014).

According to the 2023 National Institute of Statistics and Census of Argentina, approximately 0.4% of the general population (i.e., 170,519 individuals) in Argentina identify as TGD individuals (Censo Nacional de Población Hogares Viviendas, 2022). TGD individuals living in Argentina often experience harsh discrimination, victimization, and marginalization (Fundación Huésped: gender identity law and transgender people access to health care in Argentina, 2014; Radusky et al., 2021; Caballero et al., 2024), which extends to the context of healthcare and has led many to distrust the health system and express unwillingness to seek health services (Frola C. E. et al., 2023; Socías et al., 2014; Zalazar et al., 2018). Despite several studies focused on TIS in Latin America and Argentina, there is a lack of validated scales specifically designed to measure gender-related stigma experienced by TGD individuals in these regions (Caballero et al., 2024; Aristegui et al., 2018; Radusky et al., 2022). Although other TIS instruments have been developed outside Latin America and Argentina, there are no TIS scales available in Spanish, and most are not validated for TGD individuals and, therefore, do not account for their lived experiences (King et al., 2020). As such, the purpose of this study was to evaluate the psychometric properties of a TIS scale among TGD individuals living in Argentina.

## 2 Materials and methods

### 2.1 Design

Psychometric validation of the TIS Scale is based on a secondary data analysis of the TransCITAR baseline data. TransCITAR is a prospective cohort study of TGD individuals living in the metropolitan area of Buenos Aires, Argentina. Details of TransCITAR have been described elsewhere (Cartujano-Barrera et al., 2023; Frola C. et al., 2023). Study procedures were approved and monitored by Fundacion Huésped’s Institutional Review Board (IRB00002014 # FH-41). Psychometric evaluation of the TIS Scale followed standards of the International Test Commission (Hernández et al., 2020). It was conducted in partnership with a Community Advisory Board (CAB) and the Community Engagement (CE) team at Fundación Huésped. The CAB and the CE team consisted of TGD individuals living in Buenos Aires, Argentina, who made sure that the scale collected information about significant and common situations for TGD individuals living in Argentina. Participation was voluntary and informed consent was obtained from each participant.

### 2.2 Participants

Participants were 484 TGD individuals living in Buenos Aires, Argentina. Participants were recruited through outreach efforts from peer navigators using community-based recruitment strategies. Individuals were eligible if they (1) self-reported a gender identity different from that commonly associated with their sex assigned at birth, and (2) were older than 18 years. Details of the recruitment have been described elsewhere (Frola C. et al., 2023). Based on the sample-to-item ratio (i.e., at least 5–10 times the number of items), our sample size was adequate for psychometric validation of the TIS scale (Radloff, 1977).

### 2.3 Instruments

The TransCITAR baseline survey included sociodemographic variables such as age, gender identity, education attainment, employment status, relationship status, and type of health insurance. Participants also completed the Center for Epidemiologic Studies Depression Scale (CES-D) (Radloff, 1977). CES-D scores ranged from 0 to 60. CES-D scores of 16 and greater indicated that participants were at risk of depression (Radloff, 1977). The CES-D has been previously used with the trans population (Aristegui et al., 2022). All assessments were completed in Spanish and were administered in-person by a trained peer navigator.

#### 2.3.1 The TIS scale

To develop the TIS scale, items from the People Living with HIV (PLHIV) Stigma Index (Global Network of People Living with HIV, International Community of Women Living with HIV/AIDS, International Planned Parenthood Federation and Joint United Nations Programme on HIV/AIDS, 2008) and the Everyday Discrimination Scale (Williams et al., 1997) were combined. The PLHIV Stigma Index and the Everyday Discrimination Scale were selected due to their broad applicability and validated psychometric properties (e.g., appropriate reliability and validity) (Lyons et al., 2022; Friedland et al., 2018; Wolfe et al., 2021; Treharne et al., 2020; Belloir et al., 2022). Items were then adapted by modifying the wording from “due to your HIV status” to “due to your trans identity.” This modification of the scale was made in collaboration with the CE team and with *Asociación de Travestis, Transexuales y Transgéneros de Argentina* (ATTA: Spanish for “Association of Transvestites, Transexuals, and Transgenders of Argentina”), and was used in prior studies with smaller samples (Radusky et al., 2022; Aristegui et al., 2022). The initial version of the scale consisted of 33 items, 15 assessed enacted TIS (e.g., “I was excluded from family activities”) and 18 assessed internalized TIS (e.g., “I felt ashamed”) in the last year. Participants used a 5-point Likert scale (*1-Never/Nunca, 2-Rarely/Raramente, 3-Sometimes/A veces, 4-Often/A menudo, to 5-Always/Siempre*) to rate frequency. Participants completed the developed TIS scale as part of the TransCITAR baseline survey.

### 2.4 Data analysis

Statistical analyses were conducted using JASP (JASP Team, 2024). No cases were dismissed from the total sample due to missing values. Frequencies were calculated for categorical variables and means and standard deviations for continuous variables. A *p* value of <0.05 was deemed statistically significant.

#### 2.4.1 Item analysis

Items analysis was conducted based on descriptive analysis (kurtosis and skewness), correlation coefficient method and Cronbach’s *α* or McDonald’s *ω* if an item was deleted. As recommended by Kline, items with kurtosis and skewness greater than 2 and −2, respectively, were removed (Hair et al., 2017; Kline, 2015). Similarly, as recommended by Henrysson, items with item-total correlations less than 0.3 were removed (Henrysson, 1963). Overall, this process was collaboratively reviewed by the CAB and the CE team at Fundación Huésped. The CAB and the CE team discussed each item to decide whether to include them in the scale.

#### 2.4.2 Construct validity

The Keyser-Meyer-Olkin (KMO) test of sampling adequacy and Bartlett’s Test of Sphericity were performed to verify if the collected data met the requirements to perform a factor analysis (Kaiser, 1974; Bartlett, 1937). To assess construct validity, an exploratory factor analysis (EFA) of the TIS scale was carried out using a principal component method with promax rotation to identify the underlying factors of the scale (Tinsley and Tinsley, 1987). An EFA was selected because this study represents the first examination of the psychometric properties of this TIS scale. Additionally, the analysis was exploratory to facilitate the identification of items to accurately measure the TIS construct (Worthington and Whittaker, 2006). Items with factor loadings lower than 0.60 were removed (Finch, 2020).

#### 2.4.3 Reliability

Cronbach’s alpha (*α*) and McDonald’s omega (*ω*) were used to evaluate the internal consistency reliability of the scale. Adequate internal consistency was defined by a Cronbach’s *α* as well as a McDonald’s *ω* of > 0.7 (Everitt, 1975; Peter et al., 2017; Heale and Twycross, 2015).

#### 2.4.4 Criterion validity

Lastly, the criterion validity of the TIS scale was explored through correlational analysis with a self-report measure of depression (Radloff, 1977; Kline, 2015)—a construct theoretically linked to stigma. Spearman’s *ρ* was used to measure correlations between TIS and depressive symptoms.

## 3 Results

The mean age of participants was 31.5 (SD 9.2). The sample consisted of 404 transgender women (83.50%), 49 transgender men (10.10%), and 31 non-binary individuals (6.40%). More than half (62.60%) of the participants had attained a secondary level of education or less. Most participants did not have an intimate partner (99.20%) and did not have formal employment (70.90%). Most participants (72.30%) did not report migrating to or from the country, however, almost half of them indicated migrating internally across Argentina (43.60%). Almost half of the participants were currently engaged in sex work (47.11%). Other characteristics are described in Table 1.

**Table 1:** Sociodemographic characteristics of the participants (*n* = 484).

Characteristic	*N* (%)
Age, Mean (SD)	31.5 (9.2)
Gender identity	
Transgender women	404 (83.50%)
Transgender men	49 (10.10%)
Non-binary	31 (6.40%)
Education attainment	
Primary education or less	42 (8.68%)
Secondary education or less	303 (62.60%)
Post-secondary education	139 (28.72%)
Employment status	
Employed	141 (29.13%)
Unemployed	343 (70.87%)
Relationship status	
Has intimate partner	4 (0.83%)
Single	480 (99.17%)
Migrant status	
Yes	134 (27.70)
No	350 (72.30%)
Internal migrant	
Yes	211 (43.60%)
No	139 (28.70%)
Not applicable	134 (27.70%)
Current sex work	
Yes	228 (47.11%)
No	256 (52.89%)

Of the 33 items in the TIS scale, 19 items were considered for removal because of their skewness and/or kurtosis. Moreover, item 29 was considered for removal due to poor correlation. Despite their skewness and kurtosis, the CAB decided to include items 8, 9, 16 and 18 because they collected information about significant and common situations for TGD individuals living in Argentina (Table 2).

**Table 2:** Descriptive analysis of the TIS scale items for total sample (*n* = 484).

Scale items	Variance	Skewness	Kurtosis	Corrected item-total correlation	Cronbach’s α if item deleted	McDonald’s ω if item deleted	Decision
Due to your trans identity, in the last year, how often did the following situations happen to you in your daily life? / *Por tu identidad trans ¿en el último año, con qué frecuencia te sucedieron las siguientes situaciones en tu vida cotidiana?*							
Enacted TIS							
1. People treat you less politely *La gente te trata con menos cortesía*	0.89	1.81	3.22	0.62	0.86	0.88	Retained
2. People treat you with less respect *La gente te trata con menos respeto*	0.89	1.71	2.76	0.63	0.86	0.88	Retained
3. You receive less than adequate service (in a business, restaurants, etc.) *Recibís un servicio menor al adecuado (en un negocio, restaurante, etc.)*	0.88	1.88	3.29	0.53	0.86	0.88	Retained
4. People act like they are afraid of you *La gente actúa como si te tuvieran miedo*	0.55	2.91	9.41	0.59	0.86	0.88	Deleted
5. People act like you are not smart *La gente actúa como si no fueras inteligente*	1.0	2.13	4.05	0.60	0.86	0.88	Retained
6. People act like you are dishonest *La gente actúa como si fueras deshonesta/o*	0.87	2.36	5.33	0.56	0.86	0.88	Retained
7. People act like they are better than you *La gente actúa como si fueran mejores que vos*	1.3	1.68	1.95	0.69	0.86	0.87	Retained
8. People insult you *La gente te insulta*	0.83	2.21	4.71	0.57	0.86	0.88	Retained (CAB)
9. People threaten or verbally harass you *La gente te amenaza o acosa verbalmente*	0.70	2.53	6.38	0.56	0.86	0.88	Retained (CAB)
10. People make sexual advances towards you (on public transport or on the street) *La gente te hace insinuaciones sexuales (en el trasporte público o en la calle)*	1.5	1.63	1.43	0.51	0.86	0.88	Retained
11. You were excluded from social gatherings or activities (such as weddings, funerals, parties, clubs) *Fuiste excluida/o de reuniones o actividades sociales (ej. bodas, funerales, fiestas, clubes)*	0.34	4.31	19.95	0.45	0.87	0.88	Deleted
12. You were excluded from religious activities/places of worship *Fuiste excluida/o de actividades religiosas o de lugares de culto*	0.27	6.59	44.77	0.24	0.87	0.88	Deleted
13. You were excluded from family activities (e.g., cooking, eating together, sleeping in the same room) *Fuiste excluida/o de actividades familiares (ej: cocinar, comer juntos, dormir en la misma habitación)*	0.94	3.27	9.38	0.28	0.87	0.88	Deleted
14. Your partner or any members of your household experienced discrimination as a result of your trans identity *Tu pareja o alguno de los miembros de tu grupo familiar experimentó discriminación como resultado de tu identidad trans*	0.39	4.21	19.46	0.40	0.87	0.88	Deleted
15. You experienced sexual rejection from a potential partner as a result of your trans identity *Experimentaste rechazo sexual por parte de una potencial pareja como resultado de tu identidad trans*	0.22	4.29	22.14	0.37	0.87	0.88	Deleted
Internalized TIS							
16. You felt ashamed *Te sentiste avergonzada/o*	0.39	3.11	10.89	0.33	0.87	0.88	Retained (CAB)
17. You felt guilty *Te sentiste culpable*	0.22	4.77	28.16	0.33	0.87	0.88	Deleted
18. With low self-esteem *Con baja autoestima*	0.66	2.60	6.78	0.49	0.86	0.88	Retained (CAB)
19. You feel like you should be punished *Sentís que deberías ser castigada/o*	0.02	8.90	88.27	0.09	0.87	0.88	Deleted
20. You had suicidal thoughts *Tuviste ideas suicidas*	0.17	5.29	34.32	0.39	0.87	0.88	Deleted
21. Being around other trans people makes me uncomfortable *Estar cerca de otras personas trans me incomoda*	0.54	3.39	12.45	0.26	0.87	0.88	Deleted
22. I would prefer not to be trans *Preferiría no ser trans*	0.43	−4.64	22.45	0.21	0.87	0.88	Deleted
23. I feel comfortable being trans *Me siento cómoda/o siendo trans*	1.52	−1.78	1.81	0.06	0.88	0.89	Deleted
24. Even if I could change my gender identity, I would not do it *Aunque pudiera cambiar mi identidad de género, no lo haría*	2.31	−1.56	0.56	−0.31	0.89	0.90	Retained
25. You preferred not to attend social gatherings *Preferiste no asistir a reuniones sociales*	0.53	3.17	10.60	0.42	0.87	0.88	Deleted
26. You isolated yourself from your family *Te aislaste de tu familia*	1.78	1.73	1.40	0.41	0.87	0.88	Retained
27. You isolated yourself from your friends *Te aislaste de tus amigos*	0.57	3.67	13.47	0.41	0.87	0.88	Deleted
28. You stopped taking public transportation *Dejaste de tomar transporte público*	0.45	4.34	19.16	0.41	0.87	0.88	Deleted
29. You did not return to your city/hometown *No volviste a tu ciudad/pueblo de origen*	2.03	1.81	1.42	0.15	0.88	0.89	Deleted
30. Fear of being the target of gossip *Temor a ser blanco de murmuraciones*	1.15	1.98	3.10	0.60	0.86	0.87	Retained
31. Fear of being insulted, harassed and/or verbally threatened *Temor a ser insultada/o, acosada/o y/o amenazada/o verbalmente*	1.10	1.88	2.91	0.66	0.86	0.87	Retained
32. Fear of being physically attacked *Temor a ser agredida/o físicamente*	1.21	1.78	2.33	0.62	0.86	0.87	Retained
33. Fear that someone would not want to engage in an intimate sexual relationship with you because of your trans identity *Temor a que alguien no quiera entablar una relación sexual íntima con vos debido a tu identidad trans*	0.75	2.18	4.74	0.56	0.86	0.87	Deleted

### 3.1 Construct validity

After item removal, an EFA was conducted with the remaining 15 items (Table 3). The KMO measure of sampling adequacy was 0.82, which confirmed factorability of the TIS scale correlation matrix. Moreover, the Bartlett’s Test of Sphericity showed that the items were correlated (χ2 = 7,411.13, d.f. = 91, *p* < 0.001). Two factors were extracted, explaining 44 and 44% of the variance each. Preliminarily, we assigned Enacted TIS as the name of factor 1 because all items dealt with Enacted stigma (Veale et al., 2017). Similarly, the remaining items, which fell under factor 2, dealt with Internalized stigma (Austin and Goodman, 2017). As such, we assigned Internalized TIS as the name of factor 2.

**Table 3:** EFA of the 15 TIS scale items using principal component with promax rotation.

Item	Factor	
	1	2
	Enacted TIS	Internalized TIS
1. People treat you less politely	**0.95**	
2. People treat you with less respect	**0.92**	
3. You receive less than adequate service (in a business, restaurant, etc.)	**0.99**	
4. People act like you are not smart	**0.73**	
5. People act like you are dishonest	**0.88**	
6. People act like they are better than you	**0.71**	
7. People insult you	**0.84**	
8. People threaten or verbally harass you	**0.85**	
9. You felt ashamed		**0.89**
10. With low self-esteem		**0.93**
11. Even if I could change my gender identity, I would not do it		0.56
12. You isolated yourself from your family		0.33
13. Fear of being the target of gossip		**0.84**
14. Fear of being insulted, harassed and/or verbally threatened		**0.88**
15. Fear of being physically attacked		**0.86**

Factor loadings in bold indicate that the item had a loading above 0.60.

Items 11 (even if I could change my gender identity, I would not do it) and 12 (you isolated yourself from your family) were removed due to their low factor loadings. Moreover, the CAB decided to remove items 1 (people treat you less politely) and 3 (you receive less than adequate service) due to redundant content with item 2 (people treat you with less respect). The CAB also suggested that item 10 (people make sexual advances towards you) from the original 33-item scale be included because it collected information about a significant situation for TGD individuals living in Argentina. No items were removed due to their Cronbach’s *α* or McDonald’s *ω*.

After item revision, a second EFA was conducted with the remaining 12 items (Table 4). The KMO measure of sampling adequacy was 0.83, which confirmed factorability of the TIS scale correlation matrix. Moreover, the Bartlett’s Test of Sphericity showed that the items were correlated (χ2 = 5,901.26, d.f. = 66, *p* < 0.001). Two factors were extracted, explaining 41 and 31% of the variance, respectively. The items in the second EFA fell within the same two factors, and we chose to leave the factor names assigned in the first EFA. Furthermore, the two factors were moderately correlated (*r* = 0.46, *p* < 0.05), making it possible to combine scores from both subscales and interpret a total TIS score. Table 4 presents the loadings of the second EFA.

**Table 4:** Second EFA of the 12 TIS scale items using principal component with promax rotation.

Item	Factor	
	1	2
	Enacted TIS	Internalized TIS
1. People treat you with less respect	0.78	
2. People act like you are not smart	0.84	
3. People act like you are dishonest	0.94	
4. People act like they are better than you	0.79	
5. People insult you	0.86	
6. People threaten or verbally harass you	0.90	
7. People make sexual advances towards you (on public transport or on the street)	0.70	
8. You felt ashamed		0.85
9. With low self-esteem		0.83
10. Fear of being the target of gossip		0.84
11. Fear of being insulted, harassed and/or verbally threatened		0.88
12. Fear of being physically attacked		0.82

### 3.2 Internal consistency reliability

Cronbach’s α coefficient was 0.88 for the total scale, 0.88 for the enacted TIS subscale, and 0.85 for the internalized TIS subscale. Similarly, McDonald’s ω was 0.89 for the total scale, 0.89 for the enacted TIS subscale, and 0.88 for the internalized TIS subscale.

### 3.3 Criterion validity

Statistically significant correlations were observed between the total TIS scale, the enacted TIS subscale, and the internalized TIS subscale with depressive symptoms (CES-D). Depressive symptoms showed positive and moderate associations with the total TIS scale (*r* = 0.48, *p* < 0.001), the enacted (*r* = 0.43, *p* < 0.001), and the internal TIS scale (*r* = 40, *p* < 0.001).

## 4 Discussion

This study outlined the development and evaluation of the psychometric properties of the first TIS Scale in Argentina. Standardized indicators of internal consistency reliability, construct validity, and criterion validity support the use of the TIS scale among TGD individuals living in Argentina. This validated scale could be used to measure TIS in both research and clinical settings.

The KMO showed that there was an adequate sample size for the validation of the TIS scale. Similarly, the Bartlett’s Test of Sphericity result showed that the scale obtained good construct validity. Two factors were extracted—enacted and internalized TIS. Factor 1, *Enacted TIS*, included external stimuli related to mistreatment by other people towards the TGD individual due to their gender identity. Factor 2, *Internalized TIS*, included internal stimuli related to the negative feelings and thoughts of the TGD individual related to their gender identity, which extends to anticipation of discrimination experiences. These two factors were confirmed by high factor loadings. In practice, scores for both subscales could be combined to interpret a *Total TIS* score. This two-factor structure aligns with other validated transgender identity stigma scales, which have also derived two factors in their factor analyses (Chakrapani et al., 2017; Rendina et al., 2020). However, these scales collected data on different factors, such as felt normative and anticipated stigma (Chakrapani et al., 2017; Rendina et al., 2020). In one study, *felt normative* stigma referred to awareness of societal norms that devalue certain identities (Chakrapani et al., 2017), however, the items in our TIS scale were not intended to measure awareness of discriminatory norms or felt normative stigma. In the other study, *anticipated stigma* was the persistent expectation that stigmatizing events will occur (Rendina et al., 2020). Given that some of the items in the present scale refer to the anticipation of an external stimulus (e.g., items 10–12), this difference between scales could be attributed to the fact that, in our scale, anticipation represents an internal process of the individual, and therefore, constitutes internalized stigma. In short, while other scales found in the literature collected stigma experiences related to either life in society or internal processes of the TGD individual, our scale stands out by incorporating both at the same time. Nonetheless, previous literature on measuring stigma recommends that instruments capture multiple dimensions of the construct, which includes all three factors (i.e., enacted, anticipated, and internalized) (Tanner et al., 2022). Our literature review suggests that no TIS scales have been developed that include all three factors. Future studies should focus on developing an instrument that can measure these three dimensions.

This study indicated that the TIS scale has good internal consistency for all 12 items and both the enacted and internal TIS subscales. The internal consistency of our enacted and internal TIS subscales was comparable with the results of other scales that measure the same constructs [*α* = 0.75 for Enacted stigma (Chakrapani et al., 2017) and α = 0.84 for Internalized stigma (Rendina et al., 2020)]. Similar to other studies that have reported associations between stigma and depression in TGD individuals (Puckett et al., 2020), our TIS scale correlated with symptoms of depression, suggesting good criterion validity.

Of the original 33-item TIS scale, most of the items spoke of negative experiences. An attempt was made to include items with positive content (e.g., “I feel comfortable being trans” and “Even if I could change my gender identity, I would not do it”), but they did not have good statistical results. Future scales on TIS should include more items with positive content for a more comprehensive collection of information.

### 4.1 Limitations and strengths

This study has some limitations that must be considered. First, almost all participants were transgender women. However, this sample distribution does not detract from the value of the scale. Future studies should aim to have a sample of TGD individuals that appropriately includes transgender men and non-binary individuals. Second, our scale instructions state “Due to your trans identity,” which may not be necessarily applicable to non-binary individuals. Although non-binary individuals may identity as transgender, the use of the term is not universal (Fiani and Han, 2020; Barbee and Schrock, 2019). Future studies should test the equivalence of the TIS scale in non-binary individuals. Third, the sample consisted of TGD individuals in Buenos Aires, Argentina. This could lead to possible bias of experiences among TGD individuals living in urban areas. However, 43.60% of the sample were internal migrants, so we consider that representativeness is not threatened. Future studies should include TGD individuals from rural areas and/or other locations in Argentina. Lastly, the scale is based on self-reported data. As a subjective construct, stigma needs to be self-reported to be measured, which represents an inseparable limitation of any stigma instrument.

Despite these limitations, this study has some strengths that should also be considered. First, TGD individuals were included in the process of developing and validating the scale, which is significant given that other scales have not received community input. This community-engaged approach resulted in a 12-item scale with significant and common situations for TGD individuals living in Argentina, and with appropriate psychometric properties. Second, the sample size was adequate for psychometric validation. Third, the two-factor structure captures both experiences related to life in society and inner life. Lastly, this scale was developed in Spanish and validated among individuals living in a middle-income country.

## 5 Conclusion

Our validated TIS scale showed great internal consistency reliability, and construct and criterion validity among TGD individuals, with applicability in both research and clinical settings. Future research should examine other aspects of instrument validity (e.g., content and predictive validity).

## Data Availability

The original contributions presented in the study are included in the article/supplementary material, further inquiries can be directed to the corresponding author/s.
